# Assessment of consistency in contouring of normal‐tissue anatomic structures

**DOI:** 10.1120/jacmp.v4i1.2538

**Published:** 2003-01-01

**Authors:** Dawn C. Collier, Stuart S. C. Burnett, Mayankkumar Amin, Stephen Bilton, Christopher Brooks, Amanda Ryan, Dominique Roniger, Danny Tran, George Starkschall

**Affiliations:** ^1^ Department of Radiation Physics The University of Texas M. D. Anderson Cancer Center 1515 Holcombe Boulevard Houston Texas 77030

**Keywords:** contour delineation, manual segmentation

## Abstract

The purpose of this work is to estimate the uncertainty in the manual contouring of normal anatomical structures. The heart, esophagus, and spinal cord were contoured manually on six sets of computed tomography images by six dosimetrists whose experience ranged from 1 year to over 15 years. To determine the differences between inter‐ and intraobserver variations, each data set was contoured by one of the dosimetrists five times and once each by the five other dosimetrists. The magnitude of the discrepancies in delineating the contours was assessed. Intradosimetrist contouring discrepancies were as follows: esophagus, average 0.3 cm and maximum 2.9 cm; heart, average 0.5 cm and maximum 7.6 cm; and spinal cord, average 0.1 cm and maximum 0.7 cm. Interdosimetrist contouring discrepancies were as follows: esophagus, average 0.4 cm and maximum 3.1 cm; heart, average 0.7 cm and maximum 8.1 cm; and spinal cord, average 0.2 cm and maximum 0.9 cm. Significant discrepancies can occur when normal anatomic structures are contoured manually. Interdosimetrist discrepancies are typically slightly greater than intradosimetrist discrepancies. The magnitude of the discrepancies does not appear to be correlated to the experience of the dosimetrist. © *2003 American College of Medical Physics*.

PACS number(s): 87.53.–j, 87.66.–a

## INTRODUCTION

The need for manual delineation of normal‐tissue anatomic structures is a major stumbling block that impedes full automation of the radiation treatment planning process. Consequently, much effort has been put into the search for reliable ways to automate this task.^1^–^8^ An important step in the development of any automatic contouring algorithm is assessing the accuracy of the algorithm. A reasonable expectation for the accuracy of the algorithm is that it be no worse than what is manually achievable. Knowledge of the inter‐ and intraobserver variations in manual contouring can help to quantify this expectation.

Prior studies^9^‐^18^ have examined the consistency in delineation of target volumes [gross tumor volume (GTV), clinical target volume (CTV), and planning target volume (PTV)] and normal tissue anatomic structures. Not surprisingly, intraobserver variation was found to be less than interobserver variation,^9^–^13^ for example, a 5.5% volume variation as compared with a 17.5% variation.^11^ In these cases, however, delineation of the target volume was based on interpretation of the extent of tumor as displayed in an imaging study and not on the assessment of a particular anatomic structure. Prostate target volumes are notable exceptions, because the target volume is often the entire prostate.^7^,^9^,^10^ For these cases, delineation of the target volume is tantamount to delineation of the prostate, and variations in the delineated target volume are the result of different interpretations of the extent of the anatomic structure.

The purpose of this study was to assess the variations in the manual delineation of normal anatomy. Unlike previous studies,^7^,^12^,^16^,^18^ which focused on uncertainties in the total volume of an anatomic structure, the present study assesses the spatial uncertainties that arise in locating the boundaries of anatomic structures. Because beam geometries and treatment portals determined during treatment planning are based partly on the location of one or more critical anatomic structures, errors in locating the boundaries of these structures may have profound effects on the design of a treatment plan.^19^

## METHODS AND MATERIALS

### A. Contour delineation

Six dosimetrists, with experience ranging from 1 year to over 15 years, participated in the study. The heart, esophagus, and spinal cord were outlined on computed tomography (CT) image data sets of six patients with thoracic cancer who were treated at The University of Texas M. D. Anderson Cancer Center. The CT images were acquired using a virtual simulator (AcQSim; Philips Radiation Oncology Systems, Highland Heights, OH) with a slice thickness of 0.3 cm, and between 64 and 160 transverse slices per patient data set, and then transferred to a treatment planning system (Pinnacle^3^; Philips Radiation Oncology Systems, Milpitas, CA). All patient‐identifying information was removed from the system.

To determine the intraobserver variation, each dosimetrist was assigned one of the CT data sets and asked to contour the three structures five times. Intervals between contouring sessions varied from sequentially up to several days. To determine the interobserver variation, each CT data set was contoured once each by the remaining five dosimetrists. The dosimetrists were asked not to use automatic contouring tools provided by the treatment‐planning system, but rather to contour each structure manually.

To facilitate the analysis, the contours were transferred from Pinnacle^3^ to a treatment planning system that was developed in‐house^20^ using the Radiation Therapy Oncology Group (RTOG) transfer utility provided by the Pinnacle^3^ system. The contours for each of the six patients were then separated and individual files created for each contour of the heart, esophagus, and spinal cord. A program was written to read in the contour data and carry out the analysis.

### B. Data analysis

**Figure 1:**
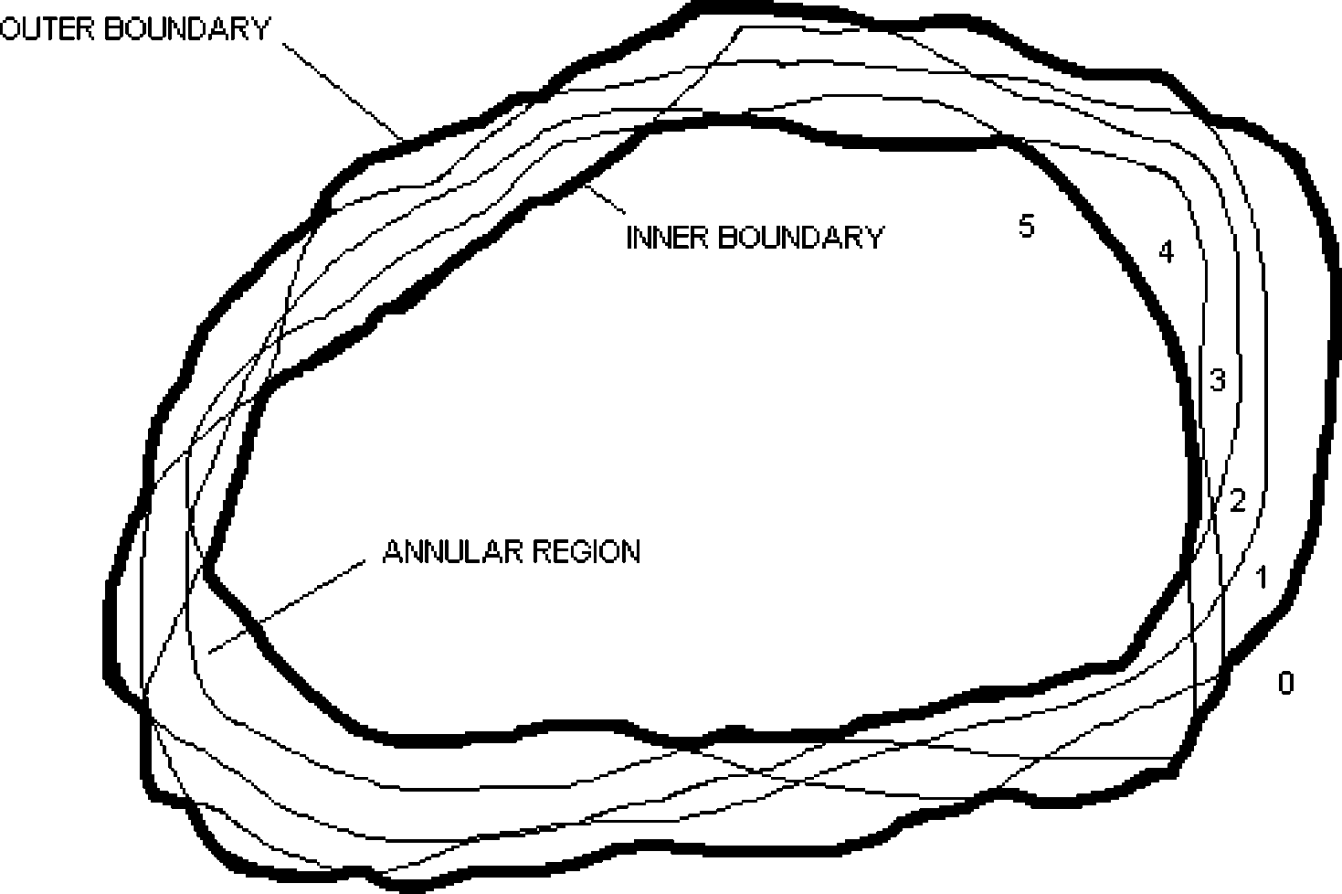
Sketch of five hypothetical contours (thin lines) that divide the plane into an inner region of points contained within all contours, an outer region of points outside all contours, and an annular region of points inside some contours and not others. The numbers 0–5 indicate the number of enclosing contours. The inner and outer boundaries are drawn with a thick line for emphasis.

If the variation in a set of contours is not too great, the contours will typically divide the plane (i.e., a particular CT slice) into three regions. These regions are an inner region of points enclosed by all contours, an outer region of points that are outside all contours, and an annular region of points that are inside some contours and not others. Figure 1 illustrates this situation for five hypothetical contours (thin lines) where, for emphasis, the inner and outer boundaries of the annular region are drawn with a thick line.

In our analysis, we examined the width of the annular region at points along its perimeter. These points corresponded to the points defining the anatomic regions on the treatment planning system and varied from around 10 to over 100 points per slice. The width can be determined from a map where each pixel is assigned a value equal to the number of contours that enclose it. A pixel is assigned a value of 5 (for a set of five contours) if it lies inside the inner region, a value of 0 if it lies in the outer region, or an intermediate value equal to the number of enclosing contours if it lies within the annular region. The map is then used to generate a histogram of the number of pixels, or area *A*(*n*), contained within *n* or more contours. Thus, *A*(5) is the number of pixels enclosed by the inner boundary, *A*(4) is *A*(5) plus the number of pixels with a value of 4, and so forth, with *A*(1) being the number pixels enclosed by the outer boundary. The quantity *A*(5)/*A*(1) equals the ratio of areas enclosed by the inner and outer boundaries.

To determine the width of the annular region from the map, we use an edge‐detection algorithm^1^ to trace out the boundary of a region with a specified pixel value. We select a threshold pixel value of 5 to trace the inner boundary and a threshold pixel value of 1 to trace the outer boundary. For each point on the outer boundary, we define the width of the annular region at that point to be the distance to the nearest inner boundary point. From this set of width “measurements” for a given anatomical structure, CT slice, and inter/intradosimetrist data set, we extract the maximum values, values corresponding to the 95th percentile width, and mean values.

## RESULTS

## A. Comparison of intradosimetrist contours

Table I summarizes for each dosimetrist, the maximum, 95th percentile, and mean discrepancies for the heart, esophagus, and spinal cord. The experience of each dosimetrist is also noted. The esophagus had a mean discrepancy of 0.3 cm and a maximum of 2.9 cm. For the heart, the mean discrepancy was 0.5 cm, and the maximum was 7.6 cm. The mean discrepancy for the spinal cord was 0.1 cm, whereas the maximum was 0.7 cm.

**Figure 2:**
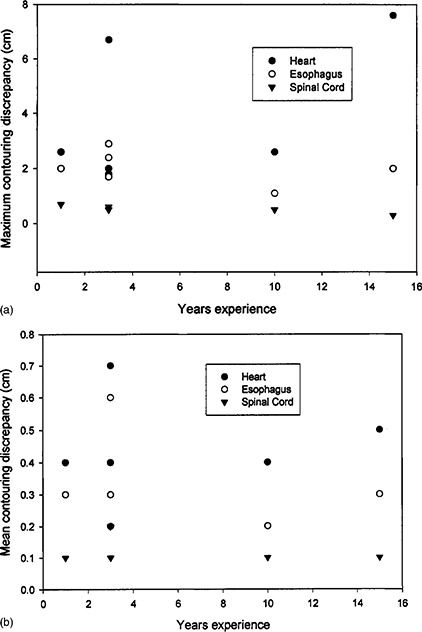
(a) Maximum discrepancy in contouring the heart, esophagus, and spinal cord for six dosimetrists with varying numbers of years of experience. (b) Mean discrepancy in contouring the heart, esophagus, and spinal cord for six dosimetrists with varying numbers of years of experience.

Figure 2 summarizes the information in Table I on graphs of maximum discrepancy versus years of experience [(Fig. 2a)] and mean discrepancy versus years of experience [(Fig. 2b)]. It does not appear that contouring discrepancy can be correlated with the experience of the dosimetrist.

In addition to the discrepancies in locating the boundaries of structures on individual transverse slices, discrepancies also arose in locating the boundaries of structures in the superior‐inferior direction. In particular, the heart is a structure that is displayed in only a limited number of slices in the thorax. Significant inconsistency existed as to the extent of the heart, even when the same dosimetrist repeated contouring the heart on the same CT data set. Table II summarizes the intra‐dosimetrist discrepancy in contouring the heart, for which variations of over 2 cm were found in the superior‐inferior direction.

**Table I:** Maximum and mean discrepancies in delineation of three anatomic structures for each of the six dosimetrists.

Dosimetrist (experience)	Anatomic structure	Maximum (cm)	95th percentile (cm)	Mean (cm)
1	Heart	2.6	2.4	0.4
(1yr)	Esophagus	2.0	1.9	0.3
	Spinal cord	0.7	0.4	0.1
2	Heart	2.0	1.7	0.4
(3yr)	Esophagus	2.4	1.7	0.6
	Spinal cord	0.5	0.4	0.1
3	Heart	2.6	2.4	0.4
(10 yr)	Esophagus	1.1	0.8	0.2
	Spinal cord	0.5	0.4	0.1
4	Heart	1.8	1.7	0.3
	Esophagus	1.7	1.0	0.2
	Spinal cord	0.6	0.4	0.2
5	Heart	6.7	6.5	0.7
(3yr)	Esophagus	2.9	2.4	0.3
	Spinal cord	0.6	0.4	0.1
6	Heart	7.6	5.1	0.5
(15 yr)	Esophagus	2.0	1.3	0.3
	Spinal cord	0.3	0.3	0.1
All dosimetrists		Maximum	Maximum of 95th percentile	Mean
	Heart	7.6 cm	6.5 cm	0.5 cm
	Esophagus	2.9 cm	2.4 cm	0.3 cm
	Spinal cord	0.7 cm	0.4 cm	0.1 cm

### B. Comparison of interdosimetrist contours

**Figure 3:**
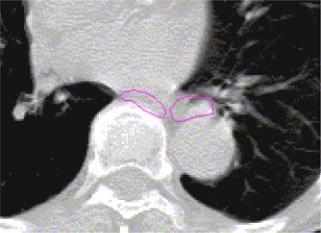
(Color) A transverse plane showing the purported esophagus outlined by two different dosimetrists. Observe that there is no overlap between the esophagus as delineated by the two dosimetrists.

Generally, interdosimetrist discrepancies were slightly greater than intradosimetrist discrepancies. The distances between the innermost contour and outermost contour varied depending on the structure. Each average distance was calculated for every slice contained in the image set. Table III summarizes the maximum and mean discrepancies for the three anatomic structures for each patient. Perhaps more remarkable than the magnitude of the discrepancies is the observation that in some transverse planes for some patients there was significant disagreement as to the location of the esophagus. Figure 3 shows an instance in which two different dosimetrists placed the esophagus in two disjoint locations. In addition, variations of over 5 cm were observed in the extent of the delineated heart in the superior‐inferior direction.

## DISCUSSION

Previous studies have demonstrated that the delineation of target volumes by radiation oncologists is highly subjective and may be significantly affected by the availability of additional information, e.g., from imaging studies. By contrast, delineation of normal anatomic structures is usually based on CT data alone and, therefore, should be somewhat less subjective. This study has found dependence on the judgment of the dosimetrist in contouring normal anatomic structures. To improve on those inconsistencies would be very desirable. Thus, an automated method should have a consistency at least as good as that which dosimetrists can achieve manually.

Agreement does not yet exist on a suitable metric for assessing consistency in contouring normal‐tissue anatomic structures. Most of the earlier studies, which, addressed contouring tumor volumes, used the ratio of volumes as a metric.^11^,^13^,^16^ This ratio may be a useful metric when applied to the evaluation of consistency in delineation of target volumes, but the relative volumes of contoured normal‐tissue anatomic structures may not accurately reflect the clinical consequences of inconsistently delineating these structures. Recently, Cardenas *et al.* ^21^ have used a standard statistical value, ***k, that assesses the number of voxels contained within the intersections and complements between two or more structure volumes. The connection between a value of ***k and clinical consequences has not yet been established.

We propose that a distance metric may be clinically relevant for assessing contouring uniformity. During the treatment‐planning process, beam geometries and treatment portals are often determined to avoid normal‐tissue anatomic structures for which the margins can be relatively small. If the boundary contour of a critical anatomic structure is displaced from its true location, it is possible that the margin between the portal edge and the structure would be insufficient to spare irradiation of the anatomic structure. This may have significant consequences for the potential morbidity of the treatment plan.

**Table II:** Discrepancy in superior‐inferior delineation of the heart for each of the six dosimetrists.

Dosimetrist	Yr experience	Discrepancy
1	1	1.2 cm
2	3	0.6 cm
3	10	1.8 cm
4	3	2.1 cm
5	3	0.6 cm
6	15	2.4 cm

The level of disagreement among dosimetrists in contouring normal‐tissue anatomy was somewhat surprising and may have significant consequences, especially for the esophagus, where, in several instances, contours were placed in disjoint locations. It is possible that, given the differences in location of the esophagus, significantly different beam configurations may be designed, leading to significant differences in the delivered dose and correspondingly different risks of morbidity.

**Table III:** Comparison of inter‐ and intradosimetrist discrepancies in delineation of three anatomic structures in transverse planes.

Heart:	Max width (cm)		Mean width (cm)	
	Inter	Intra	Inter	Intra
Patient 1	2.7	2.6	0.6	0.4
Patient 2	3.6	2.0	0.5	0.4
Patient 3	5.3	2.6	0.9	0.4
Patient 4	8.1	1.8	1.2	0.3
Patient 5	3.0	6.7	0.3	0.7
Patient 6	4.8	7.6	0.5	0.5
Esophagus:	Max width (cm)		Mean width (cm)	
	Inter	Intra	Inter	Intra
Patient 1	1.8	2.0	0.4	0.3
Patient 2	2.4	2.4	0.5	0.6
Patient 3	2.3	1.1	0.4	0.2
Patient 4	1.8	1.7	0.4	0.2
Patient 5	3.1	2.9	0.4	0.3
Patient 6	2.2	2.0	0.5	0.3
Spinal cord:	Max width (cm)		Mean width (cm)	
	Inter	Intra	Inter	Intra
Patient 1	0.8	0.7	0.2	0.1
Patient 2	0.9	0.5	0.2	0.1
Patient 3	0.8	0.5	0.3	0.1
Patient 4	0.8	0.6	0.3	0.2
Patient 5	0.5	0.6	0.2	0.1
Patient 6	0.8	0.3	0.2	0.1

Averages:	Heart		Esophagus		Spinal cord	
	Inter	Intra	Inter	Intra	Inter	Intra
Max width (cm)	4.6	3.9	2.3	2.0	0.8	0.5
Mean width (cm)	0.7	0.5	0.4	0.3	0.2	0.1

## CONCLUSIONS

Discrepancies in the manual delineation of anatomic structures can vary substantially, up to several centimeters. Generally, the discrepancies do not appear to be correlated with the experience of the dosimetrist. An automated method could standardize the delineation of anatomical structures and thereby decrease the amount of variation from patient to patient. Automated delineation would need to be at least as accurate as that achievable manually. This study will help to set a guideline for the accuracy expected of an automated method.

## ACKNOWLEDGMENT

This research was supported in part by a sponsored research agreement with Philips Radiation Oncology Systems (ADAC), Milpitas, CA.
